# Supplementary material for: The tomato receptor CuRe1 senses a cell wall protein to identify Cuscuta as a pathogen
Source: Nat Commun. 2020 Oct 20;11:5299. doi: 10.1038/s41467-020-19147-4 (PMC7576778; doi:10.1038/s41467-020-19147-4)
Supplement: Supplementary file 1 — Supplementary Information [file 41467_2020_19147_MOESM1_ESM.pdf]

Supplementary Data

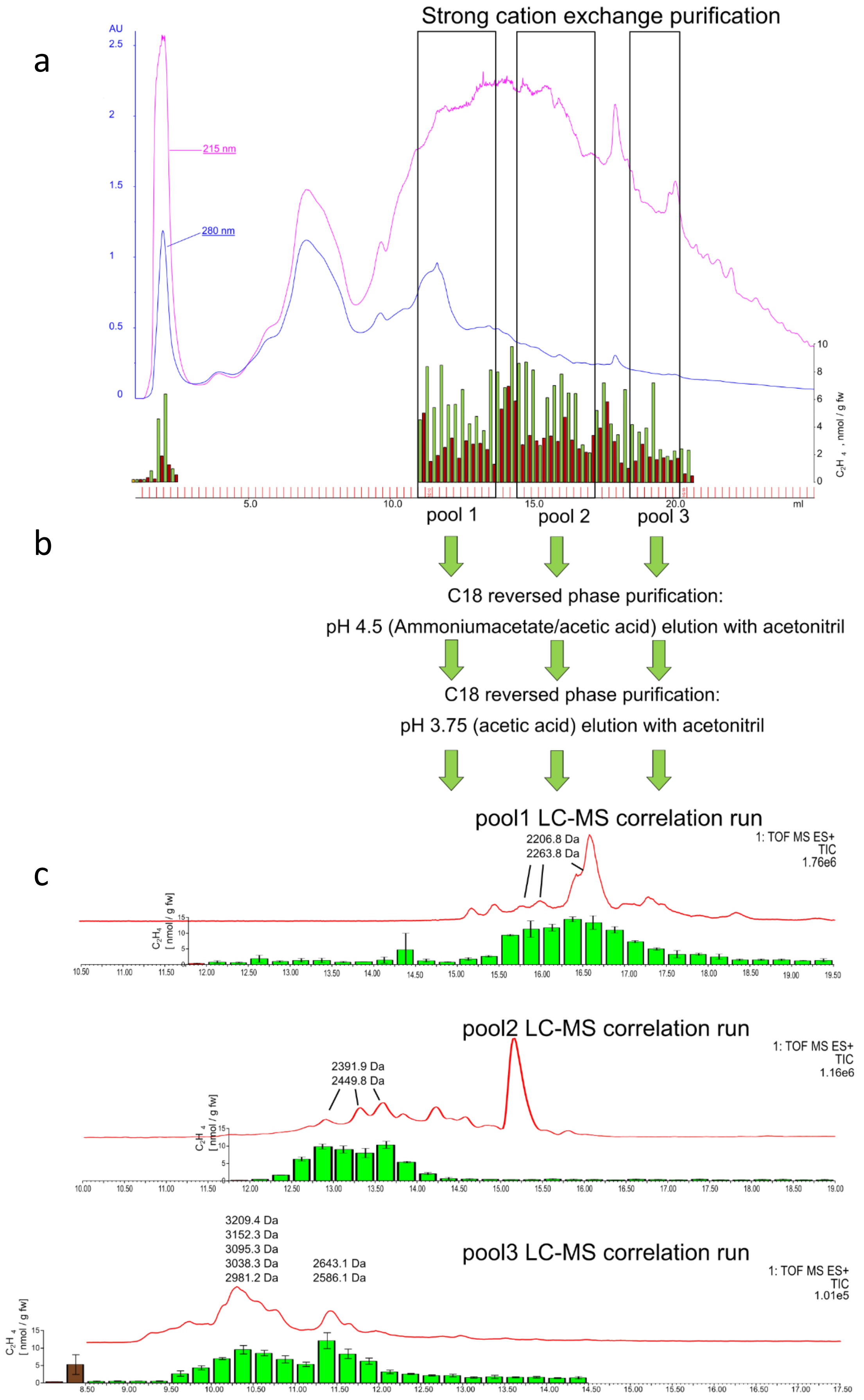
a
Strong cation exchange purification
AU
2.5
2
1.5
1
0.5
0
215 nm
280 nm
5.0
10.0
15.0
20.0
ml
10
8
6
4
2
0
C_2H_4 , nmol / g fw
pool 1
pool 2
pool 3
b
C18 reversed phase purification:
pH 4.5 (Ammoniumacetate/acetic acid) elution with acetonitril
C18 reversed phase purification:
pH 3.75 (acetic acid) elution with acetonitril
c
pool1 LC-MS correlation run
2206.8 Da
2263.8 Da
1: TOF MS ES+
TIC
1.76e6
C_2H_4 [nmol / g fw]
pool2 LC-MS correlation run
2391.9 Da
2449.8 Da
1: TOF MS ES+
TIC
1.16e6
3209.4 Da
3152.3 Da
3095.3 Da
3038.3 Da
2981.2 Da
2643.1 Da
2586.1 Da
pool3 LC-MS correlation run
1: TOF MS ES+
TIC
1.01e5

Supplementary Fig. 1. Purification of *C. reflexa* extract and identification of CuRe1 inducing molecules. (a) Separation of pre-purified *C. reflexa* extract by strong cation exchange chromatography (SCX). Eluting fractions (gradient 0-700 mM KCl; fractions in ml) were tested for induction of ethylene production in *CuRe1*-expressing *N. benthamiana*. Fractions with activity were combined to three pools as indicated. (b) Overview illustrating the subsequent purification steps on reversed phase C18 columns under two different pH regimes performed with the three pools separately. (c) Final analysis of the activities by LC-MS. Purified activities originating from pools 1, 2 and 3 from (a) were analysed with LC-MS to obtain a total ion chomatograms. In parallel runs, fractions were tested for activity in the ethylene bioassay. Bioactive peptides with masses ranging from 2206.8 Da to 3209.4 Da are indicated.

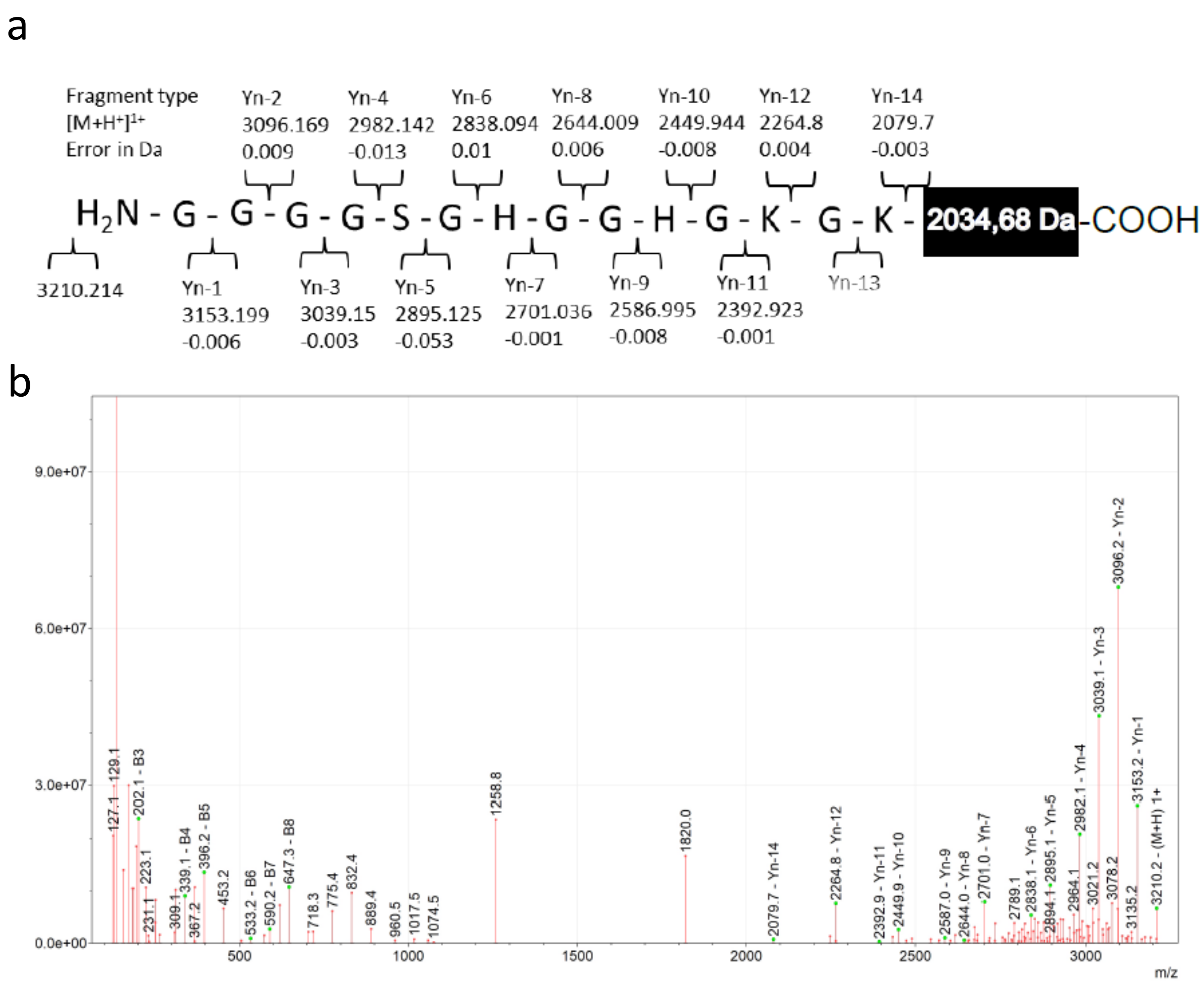

Supplementary Fig. 2. *de novo* sequenced peptide of CrGRP and corresponding MS-spectrum. (a) N-terminal peptide sequence of the Candidate mass 3209.2 Da calculated from the observed Y-fragments in the deconvoluted MS/MS spectrum; black box shows the peptide part which could not be sequenced due to inconclusive fragmentation. The mass repetitively occurred in the analyzed samples and was the responsible bioactive part (e.g. in ethylene measurements), later identified as Crip21 within the CrGRP sequence. (b). Y-fragments are labeled and indicated with green dots.

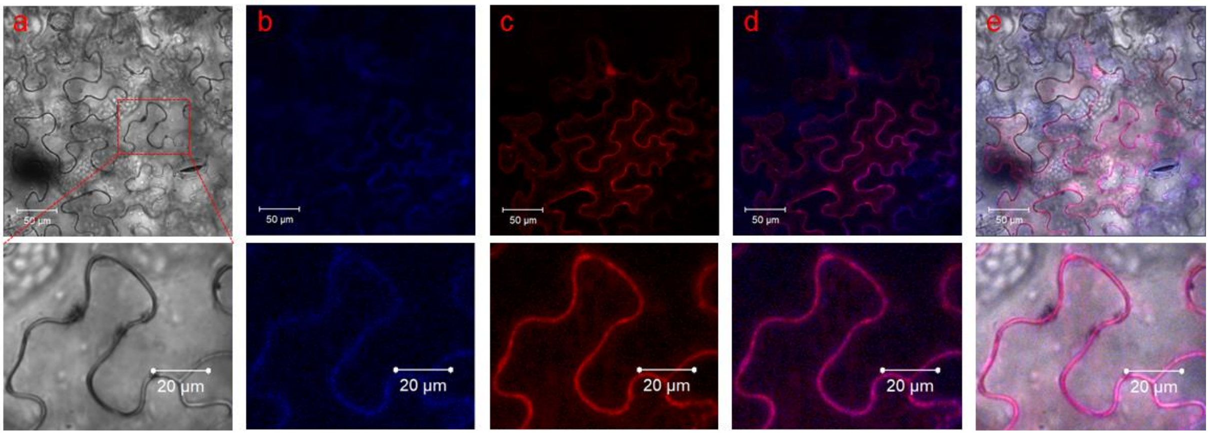

Supplementary Fig. 3. Confocal microscopy and subcellular localization of CrGRP-RFP in *N. benthamiana* leaf epidermal cells; a) bright field; b) blue: autofluorescence of the epidermal cell walls (lignin); excitation at 405 nm and emission was detected at 410-466 nm; c) Fluorescence of the RFP-tag present at the CrGRP c-terminus; excitation at 561 nm and emission was detected at 563-607 nm; d) overlay of b) and c); e) overlay of a), b) and c); lower panel: focused detail as indicated by red box in a); purple color in d) and e) arises from overlay of RFP and lignin fluorescence.

a

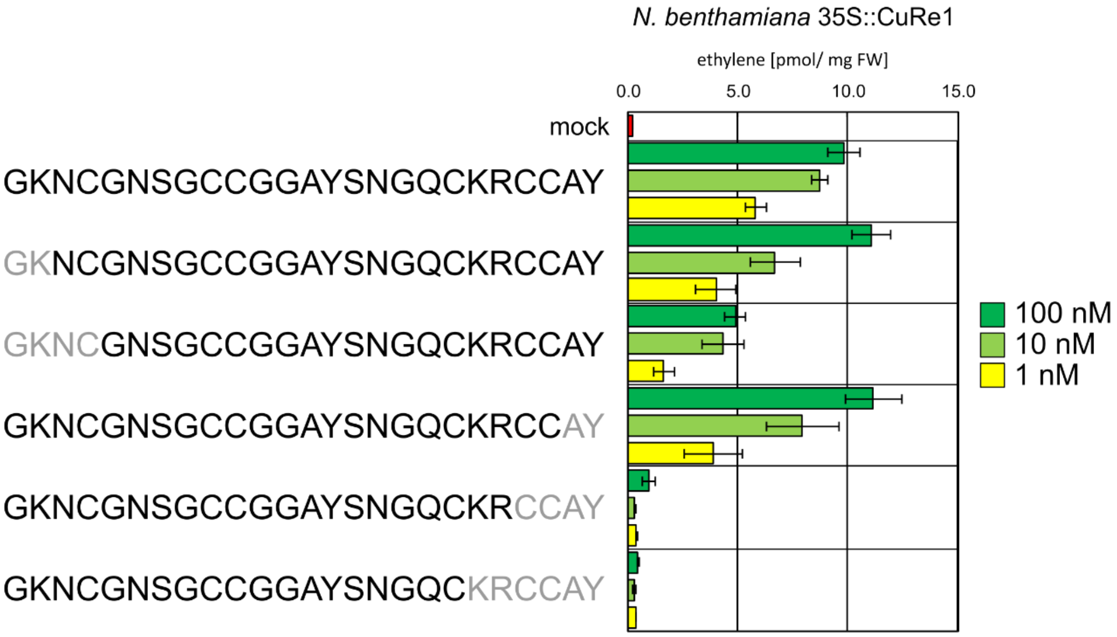

b

GRP15 GGGGSGHGGHGKGKN

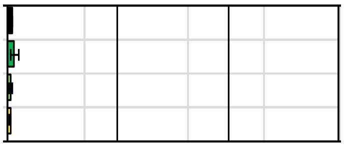

Supplementary Fig. 4. Synthesized peptides derived from CrGRP induce ethylene production in *CuRe1*-expressing *N. benthamiana*. a) The CrGRP$_{82\text{-}106}$ was n- and c-terminally truncated for finding the minimal motif triggering CuRe1; peptides were applied at the concentrations indicated. b) the peptide GRP15 which served as a fingerprint for the identification of CrGRP was completely inactive and added to the CuRe1 expressing samples at concentrations 1000, 100, 10 and 1 nM (from top). Bovine serum albumin (BSA; 0.01 mg/ml) buffered in 25 mM MES (pH 5.7) was added as mock control; FW, fresh weight; ethylene measurements show means of three technical replicates; error bars denote SD. Experiments have been repeated more than three times.

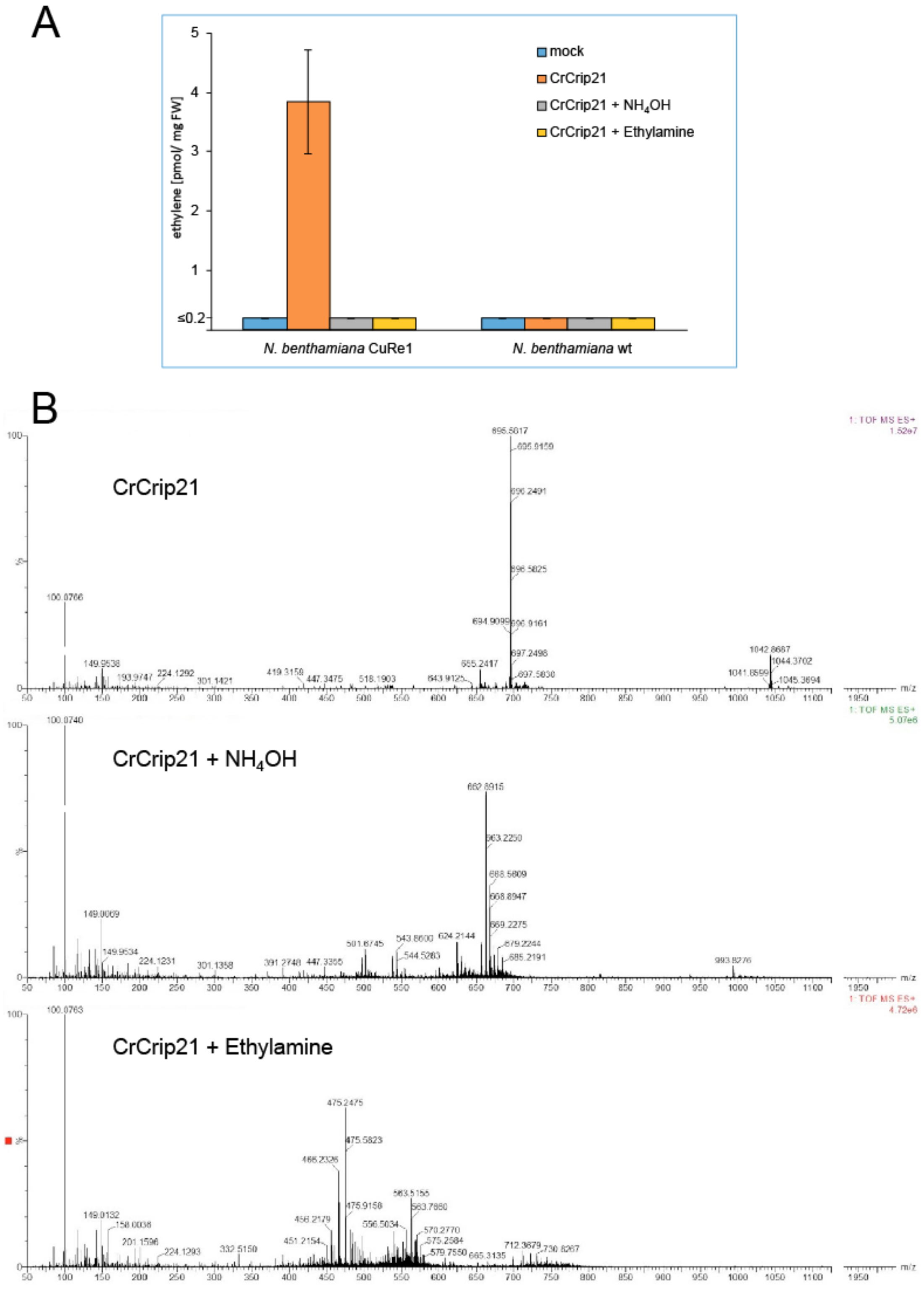

Supplementary Fig. 5. Inactivation of Crip21 by treatments with NH_4OH or Ethylamine; (a) Crip21 peptide treated with 12.5% NH_4OH or 70% Ethylamine, respectively, loose activity to induce ethylene biosynthesis in *CuRe1*-expressing *N. benthamiana* leaves. Peptide samples were applied at concentrations of 100 nM. Bovine serum albumin (BSA; 0.01 mg/ml) buffered in 25 mM MES (pH 5.7) was added as mock control. FW, fresh weight; ethylene measurements show means of three technical replicates; error bars denote SD. (b) incubation with NH_4OH or Ethylamine leads to disappearance of the peptide mass signal and appearance of multiple as yet unidentified reaction products with different masses.

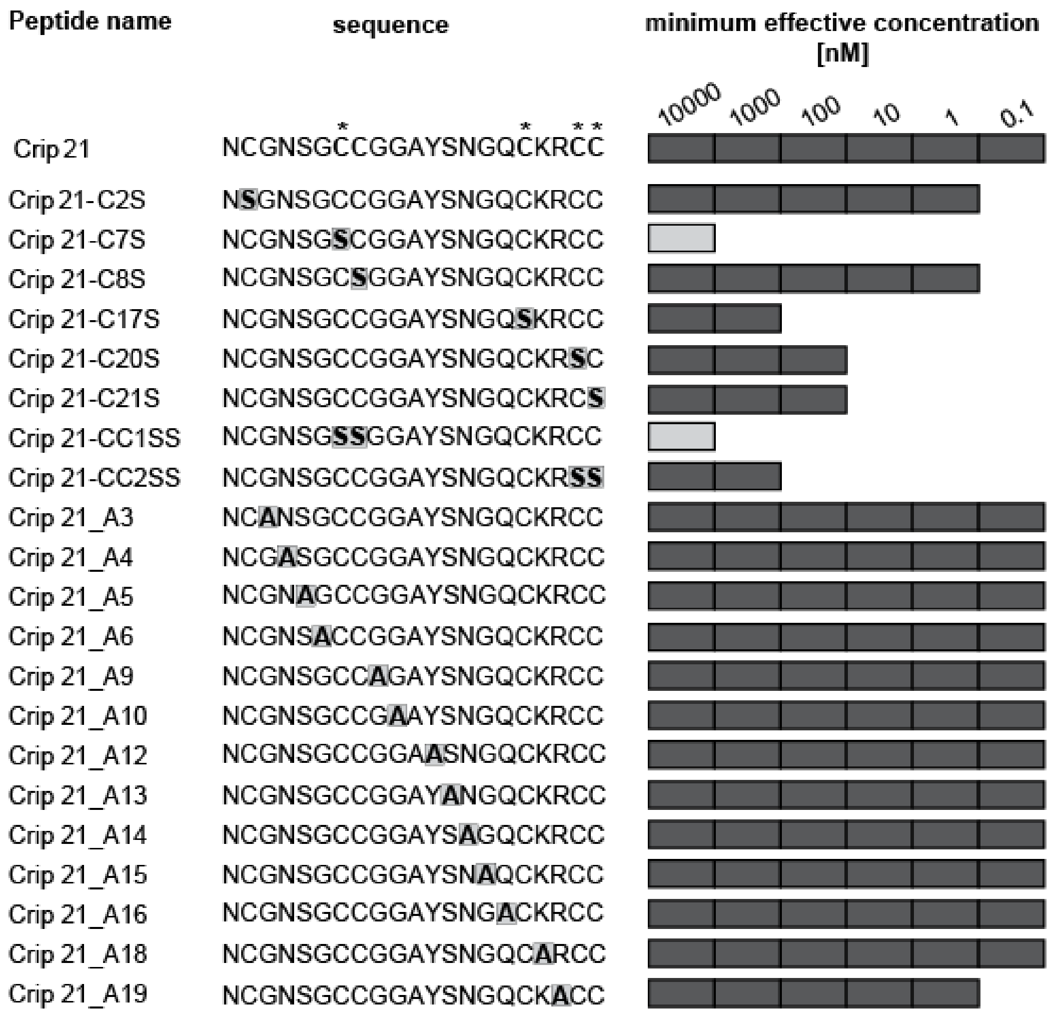

Supplementary Table 1. Activity of Crip21 peptides with substituted aa-residues; replacements of aa-residues are indicated; peptides were tested dose-dependent in ethylene bio assays using *CuRe1*-expressing *N. benthamiana* leaves (see supplemental data set s1); bars (right column) show the minimum effective concentrations; fully active peptides were similarly active as Crip21 at minimum effective concentrations of $\geq$0.1 nM; the peptides Crip21-C7S and CC1SS were both inactive at the tested maximum concentration (10,000 nM; bars in light grey). Asterisks indicate the Cysteine residues within Crip21 which are critical for full function in triggering CuRe1.

For raw data of ethylene bio-assay see also dataset s1 (.xlsx)

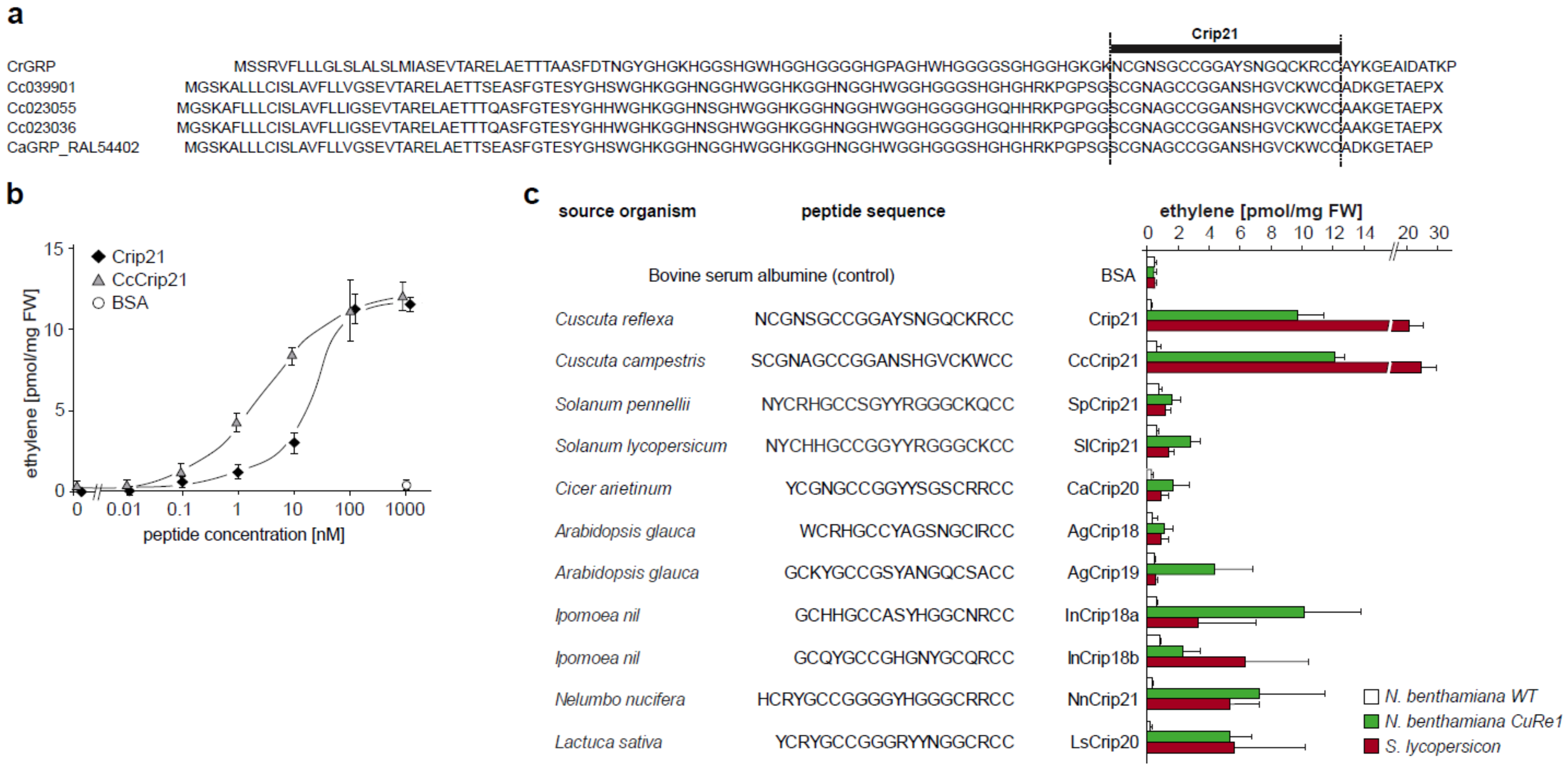
a
Crip21
CrGRP MSSRVFLLLGLSLALSLMIASEVTARELAETTTAASFDTNGYGHGKHGGSHGWHGGHGGGGHGPAGHWHGGGGSGHGGHGKGKNCGNSGCCGGAYSNGQCKRCCAYKGEAIDATKP
Cc039901 MGSKALLLCISLAVFLLVGSEVTARELAETTSEASFGTESYGHSWGHKGGHNGGHWGGHKGGHNGGHWGGHGGGSHGHGHRKPGPSGSCGNAGCCGGANSHGVCKWCCADKGETAEPX
Cc023055 MGSKAFLLLCISLAVFLLIGSEVTARELAETTTQASFGTESYGHHWGHKGGHNSGHWGGHKGGHNGGHWGGHGGGGHGQHHRKPGPGGSCGNAGCCGGANSHGVCKWCCAAKGETAEPX
Cc023036 MGSKAFLLLCISLAVFLLIGSEVTARELAETTTQASFGTESYGHHWGHKGGHNSGHWGGHKGGHNGGHWGGHGGGGHGQHHRKPGPGGSCGNAGCCGGANSHGVCKWCCAAKGETAEPX
CaGRP_RAL54402 MGSKALLLCISLAVFLLVGSEVTARELAETTSEASFGTESYGHSWGHKGGHNGGHWGGHKGGHNGGHWGGHGGGSHGHGHRKPGPSGSCGNAGCCGGANSHGVCKWCCADKGETAEP
b
Crip21
CcCrip21
BSA
ethylene [pmol/mg FW]
15
10
5
0
0
0.01
0.1
1
10
100
1000
peptide concentration [nM]
c
source organism
peptide sequence
ethylene [pmol/mg FW]
0 2 4 6 8 10 12 14 20 30
Bovine serum albumine (control) BSA
Cuscuta reflexa NCGNSGCCGGAYSNGQCKRCC Crip21
Cuscuta campestris SCGNAGCCGGANSHGVCKWCC CcCrip21
Solanum pennellii NYCRHGCCSGYYRGGGCKQCC SpCrip21
Solanum lycopersicum NYCHHGCCGGYYRGGGCKCC SlCrip21
Cicer arietinum YCGNGCCGGYYSGSCRRCC CaCrip20
Arabidopsis glauca WCRHGCCYAGSNGCIRCC AgCrip18
Arabidopsis glauca GCKYGCCGSYANGQCSACC AgCrip19
Ipomoea nil GCHHGCCASYHGGCNRCC InCrip18a
Ipomoea nil GCQYGCCGHGNYGCQRCC InCrip18b
Nelumbo nucifera HCRYGCCGGGGYHGGGCRRCC NnCrip21
Lactuca sativa YCRYGCCGGGRYYNGGCRCC LsCrip20
N. benthamiana WT
N. benthamiana CuRe1
S. lycopersicon

Supplementary Fig. 6. Activity of Crip peptides representing GRP sequences of other *Cuscuta* species and other plant species. (a) GRP protein sequences of *Cuscuta reflexa* (first lane), *Cuscuta campestris* (Cc0…) and *Cuscuta australis* (CaGRP; lowest line); Crip21 epitope highlighted; (b) Dose-dependent measurement of ethylene in CuRe1-expressing *N. benthamiana* leaves; the Crip21 peptides of *C. reflexa* and *C. campestris* (Crip21 sequence identical to *C. australis*) were applied in doses as indicated; (c) Crip peptides of other plant species (found by BLAST search) were tested for bioactivity in the ethylene bioassay; peptides applied at 5 µM each. (b) and (c)

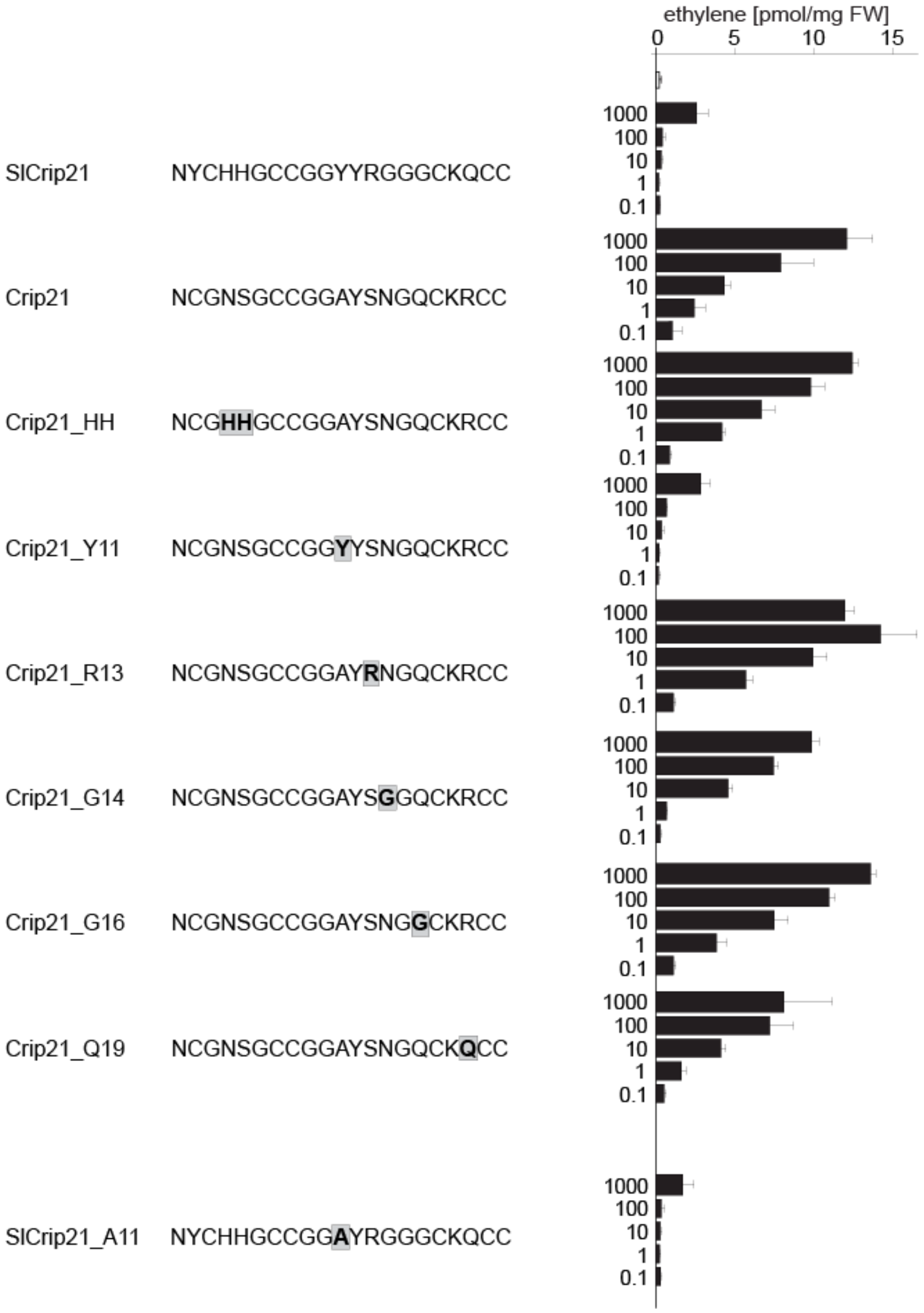

Supplementary Fig. 7 Activity of Crip21 peptides with substituted aa-residues according to the SlCrip21 sequence; replacements of aa-residues are indicated; peptides were tested dose-dependent (0.1 to 1000 nM) in ethylene bio assays using *CuRe1*-expressing *N. benthamiana* leaves; The Crip21 with a Tyrosine residue at position 11 (Crip21_Y11) was only triggering a defense response at the maximum concentration of 1000 nM and thus seems critical to abolish the peptide activity of SlCrip21. SlCrip21_A11 shows, that the single replacement of tyrosine by alanine in SlCrip21 at position 11, is not sufficient to restore the function as a defense trigger.
