# Supplementary material for: The tomato receptor CuRe1 senses a cell wall protein to identify Cuscuta as a pathogen
Source: Nat Commun. 2020 Oct 20;11:5299. doi: 10.1038/s41467-020-19147-4 (PMC7576778; doi:10.1038/s41467-020-19147-4)
Supplement: Supplementary file 3 — Reporting Summary [file 41467_2020_19147_MOESM3_ESM.pdf]

nature research

Corresponding author(s): Markus Albert

Last updated by author(s): Sep 15, 2020

Reporting Summary

Nature Research wishes to improve the reproducibility of the work that we publish. This form provides structure for consistency and transparency in reporting. For further information on Nature Research policies, see our Editorial Policies and the Editorial Policy Checklist.

Statistics

For all statistical analyses, confirm that the following items are present in the figure legend, table legend, main text, or Methods section.

n/a	Confirmed	
☐	☒	The exact sample size (*n*) for each experimental group/condition, given as a discrete number and unit of measurement
☐	☒	A statement on whether measurements were taken from distinct samples or whether the same sample was measured repeatedly
☐	☒	The statistical test(s) used AND whether they are one- or two-sided<br>*Only common tests should be described solely by name; describe more complex techniques in the Methods section.*
☐	☒	A description of all covariates tested
☐	☒	A description of any assumptions or corrections, such as tests of normality and adjustment for multiple comparisons
☐	☒	A full description of the statistical parameters including central tendency (e.g. means) or other basic estimates (e.g. regression coefficient) AND variation (e.g. standard deviation) or associated estimates of uncertainty (e.g. confidence intervals)
☐	☒	For null hypothesis testing, the test statistic (e.g. *F*, *t*, *r*) with confidence intervals, effect sizes, degrees of freedom and *P* value noted<br>*Give P values as exact values whenever suitable.*
☒	☐	For Bayesian analysis, information on the choice of priors and Markov chain Monte Carlo settings
☒	☐	For hierarchical and complex designs, identification of the appropriate level for tests and full reporting of outcomes
☒	☐	Estimates of effect sizes (e.g. Cohen's *d*, Pearson's *r*), indicating how they were calculated

Our web collection on statistics for biologists contains articles on many of the points above.

Software and code

Policy information about availability of computer code

Data collection	All data were measured as described in the Manuscript without any special software
Data analysis	obtained values have been calculated/analyzed with Microsoft Excel

For manuscripts utilizing custom algorithms or software that are central to the research but not yet described in published literature, software must be made available to editors and reviewers. We strongly encourage code deposition in a community repository (e.g. GitHub). See the Nature Research guidelines for submitting code & software for further information.

Data

Policy information about availability of data

All manuscripts must include a data availability statement. This statement should provide the following information, where applicable:

- Accession codes, unique identifiers, or web links for publicly available datasets
- A list of figures that have associated raw data
- A description of any restrictions on data availability

All data and materials will be publically available. Source data are provided with this paper. Supplementary information is available in the online version of the paper. Any other supporting data are available from the corresponding author upon request (M.A. (markus.albert@fau.de).

Field-specific reporting

Life sciences study design

All studies must disclose on these points even when the disclosure is negative.

Sample size	For (e.g. ethylene) measurements we randomly picked 3-4 leaves of different plants, cut leaves in small squares (3x3 mm), mixed them and again picked randomly 4 leaf pieces from the pool for each measurement. per treatment, 3-5 samples were measured and average inkl. SD per data-point were determined.
Data exclusions	No data were excluded
Replication	All experiments were carried out repetitively, as stated in the figure legendes (usually three times or more)
Randomization	All plants, or plant leaf material was picked randomly for all studies
Blinding	blinding was not relevant in our studies

Reporting for specific materials, systems and methods

We require information from authors about some types of materials, experimental systems and methods used in many studies. Here, indicate whether each material, system or method listed is relevant to your study. If you are not sure if a list item applies to your research, read the appropriate section before selecting a response.

Materials & experimental systems

n/a	Involved in the study	
☐	☒	Antibodies
☒	☐	Eukaryotic cell lines
☒	☐	Palaeontology and archaeology
☒	☐	Animals and other organisms
☒	☐	Human research participants
☒	☐	Clinical data
☒	☐	Dual use research of concern

Methods

n/a	Involved in the study	
☒	☐	ChIP-seq
☒	☐	Flow cytometry
☒	☐	MRI-based neuroimaging

Antibodies

Antibodies used

Primary AB;
GFP: Acris (now OriGENE) Polyclonal Antibody to GFP,
Cat. No.: R1091P
Dilution 1:5000 in 5% BSA
Host: Goat
UniProt: P42212
LOT: 27382

MYC:
Sigma Polyclonal anti-c-Myc antibody
Cat. No.: C3965
Dilution: 1:5000 in 5% BSA
Host: Rabbit
UniProt: P01106
LOT: 059M4801V

Secondary AB:
Sigma; Anti-Goat IgG (whole molecule) - Alkaline Phosphatase antibody produced in rabbit
Cat. No.: A4187
Dilution: 1:50 000 in 5% BSA
LOT: 044M4793

Sigma; Anti-Rabbit IgG (whole molecule) - Alkaline Phosphatase antibody produced in goat
Cat. No.: A3687
Dilution: 1:50 000 in 5% BSA
LOT: 129K6042

Validation

All Antibodies were used as recommended by the suppliers. (@GFP and @Myc, 1:5,000 in 5% BSA; Secondary Antibodies 1:50,000 each in 5% BSA)
